# Retraction Note: Machine learning intelligent based hydromagnetic thermal transport under Soret and Dufour effects in convergent/divergent channels: a hybrid evolutionary numerical algorithm

**DOI:** 10.1038/s41598-025-97487-1

**Published:** 2025-04-14

**Authors:** Muhammad Naeem Aslam, Nadeem Shaukat, Arshad Riaz, Ilyas Khan, Shafiullah Niazai

**Affiliations:** 1 Department of Physics and Applied Mathematics (DPAM), Pakistan Institute of Engineering and Applied Sciences, Nilore, Islamabad, 45650 Pakistan; 2 Center for Mathematical Sciences (CMS), Pakistan Institute of Engineering and Applied Sciences, Nilore, Islamabad, 45650 Pakistan; 3 Department of Mathematics, Division of Science and Technology, University of Education, Lahore, 54770 Pakistan; 4 Department of Mathematics, College of Science Al‑Zulfi, Majmaah University, 11952 Al‑Majmaah, Saudi Arabia; 5 Department of Mathematics, Education Faculty, Laghman University, Mehtarlam City, Laghman 2701 Afghanistan

Retraction of: *Scientific Reports* 10.1038/s41598-023-48784-0, published online 11 December 2023

The Editors have retracted this Article.

After publication of this Article, some of the Authors contacted the Editors to state that they had not contributed to the study. In the resulting correspondence with the Authors, the authorship of this Article could not be verified. The Editors have therefore lost confidence in the integrity of this study.

Ilyas Khan, Arshad Riaz, Nadeem Shaukat and Muhammad Naeem Aslam disagree to this retraction. Shafiullah Niazai has not stated explicitly whether they agree to this retraction.

